# The Effect of Hunger on the Likelihood of Glucose Excursions in Adults with Overweight or Obesity: Continuous Glucose Monitoring and Ecological Momentary Assessment Observational Study

**DOI:** 10.2196/85897

**Published:** 2026-06-10

**Authors:** Byunggul Lim, Phrashiah Githinji, Yue Liao, Jacob Szeszulski, Alexandra L MacMillan Uribe, Rebecca A Seguin-Fowler, Jane Anderson, Chad D Rethorst

**Affiliations:** 1 Institute for Advancing Health Through Agriculture, Texas A&M Agrilife Research - Dallas, 17360 Coit Rd, Dallas, TX, 75252, United States, 1 972 9529 625; 2 Department of Health & Kinesiology, University of Utah, Salt Lake City, UT, United States; 3 Department of Kinesiology, College of Nursing and Health Innovation, The University of Texas at Arlington, Arlington, TX, United States; 4 Institute for Advancing Health Through Agriculture, Texas A&M Agrilife Research, College Station, TX, United States; 5 Department of Neurology, Baylor College of Medicine, Houston, TX, United States

**Keywords:** hunger, glucose, continuous glucose monitoring, glycemic control, ecological momentary assessment

## Abstract

**Background:**

Maintaining stable glucose levels is important for metabolic health. Glucose excursions (GEs), which are marked increases in glucose following food intake, have been associated with a higher risk for cardiovascular disease and metabolic dysfunction. Individuals with overweight or obesity who do not have diabetes may still show impaired glucose regulation, as reflected in increased glucose variability. Hunger, as a real-time physiological cue, may be associated with subsequent glucose changes and represents a potential target for just-in-time adaptive interventions.

**Objective:**

This study aimed to investigate the temporal relationship between self-reported hunger and subsequent glucose dynamics, including the likelihood of GE occurring, in adults with overweight or obesity using continuous glucose monitoring and ecological momentary assessment.

**Methods:**

Data from 84 nondiabetic adults with overweight or obesity (mean age 47.7, SD 11.5 years) were analyzed over a 14-day period. Participants were recruited through community-based methods and enrolled between October 2022 and April 2023. Ecological momentary assessment reports of hunger were collected 6 times daily via smartphone prompts, and interstitial glucose levels were captured via continuous glucose monitoring. Three linear mixed effects models were applied to examine (1) the association between hunger and immediate glucose level, (2) glucose level 30 minutes after hunger, and (3) glucose trajectories from 30 to 180 minutes after hunger. In addition, a logistic mixed effects model was used to estimate the likelihood of a GE occurring within 30 to 180 minutes following a hunger report.

**Results:**

Hunger reports were associated with significantly lower glucose levels both at the time of reporting (mean difference [MD] 13.6 mg/dL, 95% CI –15.9 to –11.3; *P*<.001) and 30 minutes afterward (MD –11.0 mg/dL, 95% CI –13.4 to –8.6; *P*<.001). A significant time-by-hunger interaction revealed a progressive increase in glucose levels over 180 minutes after hunger (MD +6.7 mg/dL at 180 minutes, 95% CI +5.1 to +8.3; *P*<.001). Hunger was associated with increased odds of experiencing a GE by 23.7% at 30 to 60 minutes (odds ratio [OR] 1.24; 95% CI 1.07 to 2.38; *P*=.02), 36.1% at 60 to 90 minutes (OR 1.36; 95% CI 1.61 to 2.40; *P*<.001), and 13% at 90 to 120 minutes (OR 1.13; 95% CI 1.05 to 1.63; *P*=.01). No significant GE risk was observed beyond 120 minutes.

**Conclusions:**

Hunger was associated with an initial decrease in glucose followed by a delayed rise and heightened risk of GE within 30 to 120 minutes. These findings suggest that hunger may be a real-time behavioral signal associated with subsequent glucose changes. Although these findings should be interpreted with caution given the absence of direct measures of eating behavior, they highlight the potential relevance of hunger for future behavioral and digital health interventions.

## Introduction

Maintaining stable glucose levels is essential to metabolic health for both individuals with and without diabetes [1]. Although much of the literature has emphasized diabetes prevention and management, increasing attention is being paid to glycemic variability in nondiabetic populations [2]. Glucose excursions (GEs), characterized by fluctuations from the individual’s mean glucose level, have been linked to metabolic markers including central obesity and inflammation in nondiabetic adults [3]. This association is particularly relevant in nondiabetic populations with overweight or obesity, who tend to exhibit greater glycemic variability, suggesting impaired glucose regulation even in the absence of diabetes [4]. Emerging evidence indicates that GEs are associated with insulin resistance, inflammation, and increased cardiovascular risk [5]. Frequent GE may contribute to physiological stress and metabolic dysfunction over time, underscoring the importance of maintaining glycemic stability even in nondiabetic individuals with overweight or obesity [6].

Interventions emphasizing diet and physical activity are widely recommended to control glucose; however, these interventions often overlook the multifaceted dynamic physiological and behavioral influences on glycemic control [7,8]. Variables such as nutrient composition, meal timing, circadian rhythms, and cortisol fluctuations also have substantial impacts on glycemic levels [9]. Hunger, in particular, is a dynamic physiological signal that may be closely associated with within-day glycemic variability, illustrating the complex interplay between metabolic demands and glucose regulation [10]. Beyond metabolic cues, hunger may also be influenced by emotional or stress-related factors, further complicating its relationship with glycemic control [11]. Hunger is not only a subjective sensation but also a complex physiological signal reflecting energy needs, hormonal fluctuations (eg, ghrelin and insulin), and behavioral readiness for food intake [10]. It arises spontaneously and varies throughout the day and often precedes food consumption, positioning it as a critical trigger with downstream behavioral consequences. Despite this, the moment-to-moment glycemic responses that follow hunger, particularly in nondiabetic individuals with overweight and obesity, remain underexplored in the literature.

Although traditional self-report or observational methods have informed our understanding of glycemic patterns, they are limited by recall bias and low temporal resolution [12]. Recent advances in mobile technologies such as continuous glucose monitoring (CGM) and ecological momentary assessment (EMA) overcome these limitations by enabling real-time tracking of physiological and behavioral signals in naturalistic settings [13,14]. The use of CGM has been increasingly applied in nondiabetic populations with overweight or obesity, offering new opportunities for preventive insight [15]. However, many studies using CGM focus on static measures, overlooking how glucose fluctuates across short postbehavioral time windows (eg, 60‐180 minutes) [1,16]. Similarly, EMA has been used to explore hunger and affective states [17,18], but few studies have directly examined their moment-to-moment association with glucose levels. Some studies have used CGM and EMA to explore constructs such as mood and energy [12,19], and others have assessed energy states using these methods [19]; however, to our knowledge, no study has directly examined how self-reported hunger predicts short-term glucose variability in nondiabetic individuals with overweight or obesity.

Given the limited understanding of how hunger influences glycemic responses, this study integrated CGM and EMA to examine real-time associations between self-reported hunger and glucose in nondiabetic individuals with overweight or obesity. More specifically, our purposes were to (1) assess glucose changes immediately and up to 180 minutes following self-reported hunger and (2) determine whether hunger predicted the likelihood of subsequent GE. By focusing on these temporal patterns, this research has the potential to provide foundational evidence for the development of personalized, just-in-time adaptive interventions (JITAI) aimed at improving metabolic health.

## Methods

### Study Design

This study was a 14-day single-arm observational investigation designed to examine the relationship between real-time self-reported hunger and glucose fluctuations. The study integrated CGM and EMA technologies to capture real-time physiological and behavioral data in free-living conditions. The study protocol, including recruitment, screening, and monitoring procedures, is described in detail in the published protocol paper by Rethorst et al [20]. The protocol was approved by the Texas A&M University Institutional Review Board (IRB2021-1412F). The study procedures followed the previously published protocol, and no deviations were made.

### Participants

Inclusion criteria required individuals to be between 18 to 65 years of age, have a BMI ≥25, be fluent in English, and own a smartphone. Exclusion criteria included (1) current or prior diagnosis of type 1 or type 2 diabetes, (2) pregnancy, (3) diagnosis of a gastrointestinal or eating disorder, and (4) regular (defined as daily or near-daily) use of medications or supplements known to interfere with CGM accuracy or glucose metabolism, including corticosteroids, metformin, vitamin C, aspirin, and acetaminophen. Participants were recruited by community flyers, social media advertisements, and an email contact list consisting of community members who opted to be notified about upcoming research studies. Interested individuals completed an online screening survey consisting of health history and behavioral eligibility questions. Eligible individuals were invited to attend an enrollment visit at which eligibility was confirmed through objective measurements (eg, height and weight) and verification of self-reported medical history and medication use. All participants completed an informed consent document prior to completing any screening or baseline procedures and received up to US $71 for participation. Participants were enrolled from October 5, 2022, to April 26, 2023.

As this study was based on a previously developed observational protocol with a target sample size of 100 participants, the sample size was determined based on feasibility considerations [20]. Individuals who did not respond to recruitment efforts or declined participation were not included in the study, and reasons for nonparticipation were not systematically recorded.

### Measures

#### Demographics and Biometric Measures

Participants completed a demographics form (age, sex, race, ethnicity, and zip code) electronically using Research Electronic Data Capture (REDCap; Vanderbilt University) during their in-person baseline visit. Participants reported their biological sex (female or male) as part of the demographic assessment. Height and weight were measured using a standardized protocol. Repeated measures were taken to ensure accuracy, and the average of 2 consistent values (within 0.5 cm or 0.1 kg) was used in the analysis. BMI was calculated using the standard formula [21].

#### Continuous Glucose Monitoring

Participants wore a FreeStyle Libre Pro CGM sensor (Abbott Diabetes Care, Alameda, California, United States) on the upper arm for 14 consecutive days. The sensor collected blinded interstitial glucose data at 15-minute intervals across the entire monitoring period. CGM data were uploaded at the follow-up visit by scanning the sensor.

#### Ecological Momentary Assessments

Participants downloaded the Ethica Data mobile app (Ethica Data Services Inc., Waterloo, Ontario, Canada) at baseline. The Ethica app is a research platform that enables the collection of real-time self-report data through customizable smartphone-based EMA. Participants subsequently received 6 prompts per day during the 14-day monitoring period, randomly delivered within 6 fixed 2-hour intervals (8:00 AM-10:00 AM,..., 6:00 PM-8:00 PM). Six prompts per day were selected to balance temporal resolution with participant burden. Prompts were randomly delivered within fixed time windows to capture variability in hunger experiences across the day while minimizing predictability and reducing response bias. EMA procedures followed the protocol described in the study by Rethorst et al [20], which included repeated daily assessments of hunger (“Are you hungry right now?”). Compliance was monitored throughout the study period using the Ethica platform. Missed prompts were treated as missing data and were not readministered. All EMA responses were checked for completeness and EMA entries with invalid or missing time stamps were excluded prior to analysis. Feasibility was assessed using compliance metrics across EMA and CGM, including the proportion of completed EMA prompts and CGM device wear time throughout the study period.

### Data Preprocessing

All data were time stamped and processed using Python (version 3.11; Python Software Foundation). As the 2 data streams (CGM and EMA) differ in sampling frequency and timing, preprocessing was necessary to align CGM readings with EMA entries and construct a unified time-resolved dataset. Glucose values were extracted at the closest time point to each EMA and averaged over successive 30-minute post-EMA intervals (ie, 0‐30, 30‐60, 60‐90, 90‐120, 120‐150, and 150‐180 minutes) to model temporal changes in glucose following self-reported hunger. GE was defined as glucose fluctuations exceeding +1.5 or –1.5 SD from an individual’s mean glucose level. This threshold was selected to focus on larger, more pronounced excursions that may have greater clinical relevance [22]. GE events were identified within each post-EMA window. Hunger responses were binary-coded (“yes”=1; “no”=0), and relevant covariates such as sex, BMI, and weekday versus weekend were merged into the analytic dataset. All preprocessing and transformation were performed using standard Python-based data science libraries [23] to align and compute CGM and EMA data [24,25].

### Statistical Analysis

**Figure 1.**
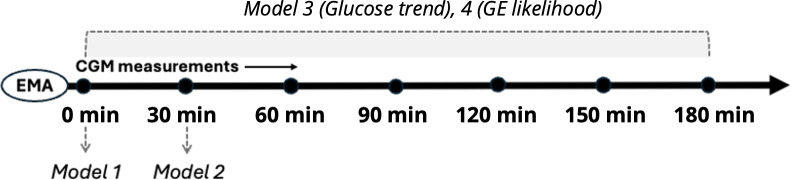
Analytic timeline of statistical models aligned to ecological momentary assessment (EMA) and continuous glucose monitoring (CGM) data. This figure illustrates the analytic time windows used for extracting CGM data relative to EMA responses across 4 statistical models. Model 1 used the CGM value closest to the EMA response, while model 2 used the 30-minute mean post-EMA. Models 3 and 4 examined trends and glucose excursion (GE) likelihood from 30 to 180 minutes.

Four mixed effects models were constructed to examine the relationship between self-reported hunger and glucose outcomes. We hypothesized that self-reported hunger would be associated with an increase in glucose level over time and an increased likelihood of a GE. All models included participant-level random intercepts to account for within-subject variability. The models included participant-level random intercepts to account for within-subject clustering; however, no explicit residual correlation structure (eg, autoregressive structure) was specified. Fixed-effect covariates included sex, BMI category, age, and weekday status. Given the relatively small number of male individuals enrolled in the study (n=13), models were not conducted separately for each sex. As illustrated in Figure 1, all models were aligned to the time stamp of the EMA response, using the closest available CGM value, which ranged from 0 to 14 minutes after each EMA prompt due to the 15-minute CGM sampling frequency. The 4 models were designed to capture distinct temporal relationships between hunger and glucose: immediate CGM, short-term mean, time-dependent trends, and finally the likelihood of GE. Missing data patterns were evaluated using Little’s test for missing completely at random.

### Model 1: Immediate Glucose Level (Closest Reading)

This linear mixed model evaluated the association between within-person and between-person hunger and the CGM value recorded closest to each EMA prompt, representing the immediate glycemic response.

### Model 2: 30-Minute Mean Glucose

This model assessed the mean glucose level across the 30-minute period following each EMA prompt to determine the short-term association of hunger with glucose.

### Model 3: Time-Based Glucose Trend

To examine time-dependent patterns of glucose regulation following self-reported hunger, this model assessed the cumulative average CGM glucose levels at 30-, 60-, 90-, 120-, 150-, and 180-minute intervals after each EMA prompt. The goal was to evaluate glucose trends over time following self-reported hunger.

### Model 4: GE Likelihood

A logistic mixed effects model with participant-level random intercepts was used to estimate the probability of experiencing a GE (as defined above) within multiple post-EMA intervals (30‐60, 60‐90, 90‐120, 120‐150, and 150‐180 minutes), with hunger as the primary predictor. Covariates included sex, BMI, age, and weekend status. All statistical models were implemented using the statsmodels package (version 0.14.0) in Python (version 3.11), and statistical significance was defined as *P*<.05. Data visualizations were created using matplotlib and seaborn [26,27]. Missing data from either EMA or CGM were ignored in the analyses, consistent with a complete case approach.

### Ethical Considerations

This study was approved by the Texas A&M University Institutional Review Board (IRB2021-1412F) on August 30, 2022. All participants provided informed consent before participating in this study. Participants received up to US $71 as compensation for their participation. All data were deidentified prior to analysis to ensure participant privacy and confidentiality.

## Results

### General Participant Characteristics

**Figure 2.**
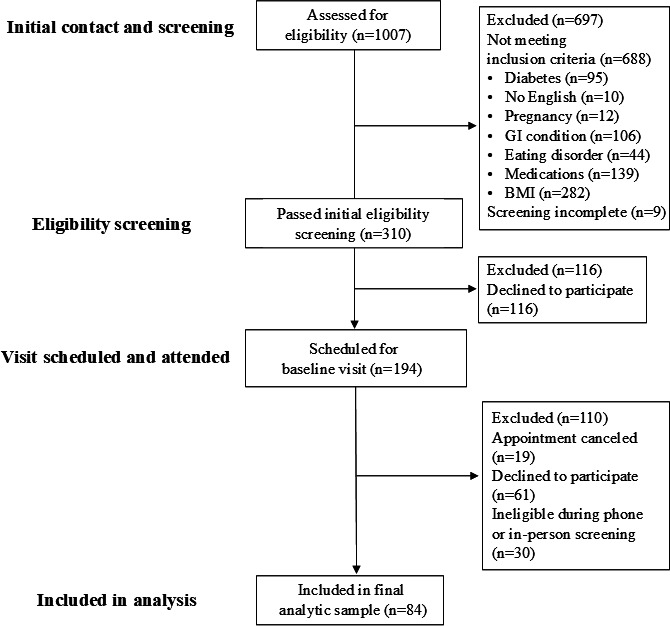
Participant flow diagram. A total of 1007 individuals completed the initial screening survey between October 2022 and April 2023. Of these, 310 passed eligibility screening, and 194 were scheduled for a baseline visit. After accounting for cancelations, ineligibility, and nonparticipation, 84 participants were enrolled and included in the final analytic sample. GI: gastrointestinal.

A total of 1007 individuals completed the screening survey. Of these, 310 passed initial eligibility screening and completed intake forms. Subsequently, 194 were scheduled for a baseline visit. However, 110 either canceled, declined further participation, or were deemed ineligible during the phone screening or at the baseline appointment. The participant flow throughout the recruitment and screening process is presented in Figure 2.

The final analytic sample included 84 participants consisting of 13 men (15.5%) and 71 women (84.5%). Demographic and biometric characteristics, including age, BMI, sex, race, and ethnicity, are summarized in Table 1. Overall compliance was moderate to high across modalities, with average adherence rates of 73% for EMA responses and 79% for CGM wear. A test of missingness using Little’s test indicated that the data were missing completely at random (*χ*²_10_=12.47; *P*=.25).

**Table 1.** Demographic and biometric characteristics of study participants.

Variables	Values
Age (years), mean (SD)	47.7 (11.5)
BMI (kg/m^2^), mean (SD)	32.8 (4.7)
Sex, n (%)	
Female	71 (84.5)
Male	13 (15.5)
Race, n (%)	
American Indian or Alaskan Native	1 (1.1)
Asian	7 (8)
Black or African American	17 (19.5)
Hispanic or Latino	11 (12.6)
Native Hawaiian or Pacific Islander	2 (2.2)
White	49 (56.3)
More than one race	3 (3.5)

### Model 1: Immediate Glucose Level (Closest Reading)

Model 1 predicted the CGM glucose value recorded immediately after each self-reported hunger entry via EMA. The linear mixed effects model showed that within-person hunger was significantly associated with lower immediate glucose levels (β=−12.5 to −14.9; *P*<.001). Between-person hunger, sex, BMI category, age, and weekday status were not statistically significant predictors. These findings are summarized in Table 2.

**Table 2.** Mixed model estimates for immediate and 30-minute post–ecological momentary assessment glucose levels.

Variables	Model 1: closest			Model 2: 30-minute mean		
	Estimate	SE	*P* value	Estimate	SE	*P* value
Intercept	110.7	4.6	<.001	108.7	4.6	<.001
Within-person hunger	−13.7	1.2	<.001	−11.0	1.2	<.001
Sex (1=female)	−6.4	3.7	.08	−6.5	3.8	.08
Between-person hunger	−4.3	5.9	.47	−3.5	6.0	.55
Age (years)	2.3	1.4	.10	2.3	1.4	.10
BMI (1=obesity)	2.5	3.0	.38	3.1	2.9	.28
Weekday (reference: weekend)	−0.01	.2	.63	−0.1	0.2	.61
Group variance^a^	124.3	1.3	—^b^	127.4	1.3	—

a Variance explained by random effects at the group level.

b Not applicable.

### Model 2: 30-Minute Mean Glucose

Model 2 assessed the average glucose level over the 30-minute period following each EMA response. The linear mixed effects model indicated a significant negative association between within-person hunger and 30-minute mean glucose levels (β=−11.0 +1.2 or −11.0 −1.2; *P*<.001), indicating lower glucose in the 30 minutes following self-reported hunger. No significant associations were found for between-person hunger, sex, BMI category, age, or weekday status. Full estimates for model 2 can be found in Table 2. As a sensitivity analysis, the models were reestimated using an autoregressive (1) correlation structure to account for temporal autocorrelation. The results were largely consistent with the primary analyses, with slight attenuation in effect sizes but no change in statistical significance. Within-person hunger remained significantly associated with lower glucose levels for both immediate glucose (β=−12.95; *P*<.001) and 30-minute mean glucose outcomes (β=−10.48; *P*<.001).

### Model 3: Time-Based Glucose Trend

Model 3 examined average CGM glucose levels across multiple time points (30‐180 minutes) following each EMA prompt. Results from a linear mixed effects model indicated that glucose levels increased over time after participants reported being hungry but remained relatively stable when they were not. A significant time × hunger interaction was observed (*P*<.001); at 180 minutes, glucose levels were estimated to be 6.68 mg/dL higher following self-reported hunger. Female sex was marginally associated with lower glucose levels (β=−7.6; *P*=.05), and glucose levels were slightly lower on weekends (β=−0.5; *P*=.04). Age showed a marginal association, whereas BMI was not a significant predictor. These results are summarized in Table 3.

**Table 3.** Mixed effects model estimates for glucose levels at 30 to 180 minutes after ecological momentary assessment^a^.

Variables	Model 3: 30-, 60-, 90-minute glucose		
	Estimate	SE	*P* value
Time point (180 min)	5.4	0.7	<.001
Time × not hungry (180 min)	−6.7	0.8	<.001
Sex (1=female)	−7.6	3.9	.05
Weekend (1=weekend)	−0.5	0.2	.04
Age	0.2	0.1	.08
BMI	0.2	0.3	.55

a Includes random intercepts for study ID. Interaction reference=hungry at 180 minutes.

**Figure 3.**
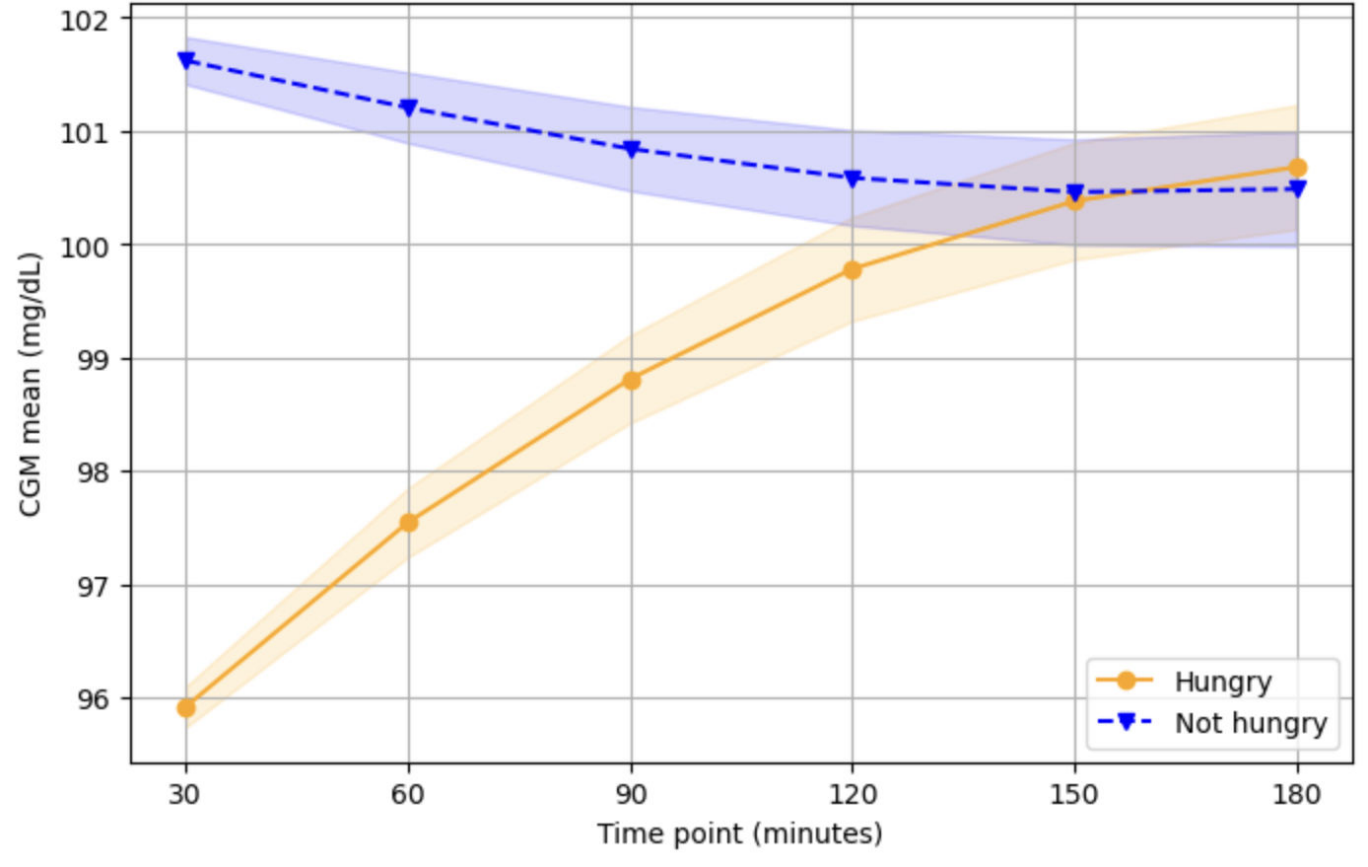
Glucose changes over time (30‐180 minutes). The figure visualizes mean glucose levels across time points (30, 60, 90, 120, 150, and 180 minutes) following ecological momentary assessment prompts. Participants who reported hunger exhibited a steady increase in glucose over time, while those who reported not being hungry maintained relatively stable or slightly decreasing levels. Shaded regions represent 95% CIs. CGM: continuous glucose monitoring.

As illustrated in Figure 3, glucose trajectories diverged over time depending on hunger status. Participants who reported being hungry showed a steady rise in glucose levels from 30 to 180 minutes, while those who were not hungry maintained relatively stable or slightly declining levels throughout the same period.

### Model 4: GE Likelihood

Model 4 analyzed the likelihood of GE occurring within 5 post-EMA time intervals: 30‐60, 60‐90, 90‐120, 120‐150, and 150‐180 minutes Table 4. Results from the logistic mixed effects regression showed that hunger was a significant predictor of GE during specific intervals. Specifically, hunger was associated with a 23.7% increase in the likelihood of experiencing a GE within 30 to 60 minutes (*P*=.02), a 36.1% increase within 60 to 90 minutes (*P*<.001), and a 13% increase within 90 to 120 minutes (*P*=.01). Hunger was not a significant predictor in the remaining intervals (0‐30, 120‐150, 150‐180 minutes; *P*>.05). Other covariates, including sex, BMI, age, and weekend status, did not show significant associations with GE occurrence across any time interval.

**Table 4.** Logistic mixed effects model results predicting glucose excursion across time intervals after ecological momentary assessment^a^.

Time interval (minutes)	Model 4: glucose excursion likelihood		
	Odds ratio (95% CI)	*P* value	Percentage change
0‐30	1.17 (0.79-2.53)	.25	+16.9
30‐60	1.24 (1.07-2.38)	.02	+23.7
60‐90	1.36 (1.61-2.40)	<.001	+36.1
90‐120	1.13 (1.05-1.63)	.01	+13.0
120‐150	1.03 (0.88-1.31)	.48	+3.3
150‐180	0.99 (0.81-1.20)	.87	–0.7

a Odds ratios reflect the likelihood of glucose excursion in the hungry condition compared to the not-hungry condition.

## Discussion

### Principal Findings

This study investigated the dynamic relationship between self-reported hunger and glucose fluctuations in nondiabetic adults with overweight or obesity using real-time data from CGM and EMA. Across 4 statistical models, results consistently demonstrated that hunger is associated with meaningful short-term changes in glycemic patterns, suggesting the potential relevance of subjective behavioral signals in metabolic regulation.

Models 1 and 2 revealed that participants demonstrate significantly lower glucose levels at the time of hunger and during the immediate 30-minute period following reported hunger. These findings align with the physiological understanding of hunger as a marker of energy deficit and a signal for imminent food intake [10]. Building on these findings, model 3 provided further evidence for this interaction by identifying a sustained increase in glucose following hunger reports, which was not observed when hunger was absent. This trend likely reflects behavioral responses to hunger, suggesting that hunger may precede food intake, which could contribute to the observed increase in glucose levels [28,29]. The progressive divergence in glucose trajectories between hungry and not-hungry states illustrates how subjective experience may precede and predict measurable metabolic changes, particularly in naturalistic, free-living environments [30]. Model 4 reinforced these findings by demonstrating that hunger significantly increased the likelihood of experiencing a GE within the 30-minute to 120-minute window after EMA prompts. The strongest association occurred during the 60-minute to 90-minute window, potentially reflecting the lag between hunger detection, food intake, and resulting glycemic response.

Overall, these findings suggest that hunger may serve as a real-time behavioral indicator associated with subsequent eating behavior, which in turn is associated with downstream glycemic fluctuations. Rather than a passive subjective state, hunger may serve as a dynamic marker of metabolic need, offering a scalable and low-burden indicator for identifying periods of glycemic vulnerability, particularly among individuals at risk for metabolic dysregulation but not yet meeting diagnostic thresholds. This insight supports the integration of CGM and EMA as a useful framework for developing JITAI that respond to momentary behavioral and physiological cues. In addition, compliance across EMA and CGM suggests that the multimodal protocol was generally feasible. However, variability in adherence and device-related issues (eg, CGM detachment) highlight potential challenges for implementation in real-world settings.

This is clinically meaningful, as GE, even in nondiabetic individuals, are increasingly linked to insulin resistance, inflammation, and cardiometabolic risk [6]. These findings suggest that hunger could be explored as a potential trigger for delivering personalized prompts targeting diet, physical activity, or stress management during high-risk windows. By showing that hunger is followed by an increased likelihood of GE, this study suggests the potential to inform future strategies aimed at guiding food choices and reducing metabolic risk. For example, hunger reports could be used to trigger JITAI that deliver tailored recommendations (eg, lower glycemic index food choices, portion control, or brief behavioral strategies) prior to eating in an effort to minimize the postprandial GE. This is clinically meaningful, as greater glycemic excursions and variability have been associated with increased oxidative stress and adverse metabolic outcomes [6,31]. In this context, hunger may function as an anticipatory behavioral cue that precedes metabolic responses, distinguishing it from physiological triggers that occur closer to or after glucose changes.

### Comparison With Prior Work

Although some prior research has explored associations between mood, stress, or energy levels and glycemic fluctuations [12,19], few have explicitly examined hunger as a time-sensitive predictor. These results thus extend earlier findings by highlighting hunger as an immediate and time-sensitive behavioral cue that may precede glycemic shifts, offering insight into real-world eating patterns [14]. The observed inverse association between concurrent hunger and glucose levels is consistent with prior physiological and behavioral research suggesting that hunger reflects a state of relative energy deficit. However, this study extends this literature by examining these relationships using high-frequency, real-time data in naturalistic settings, allowing for the characterization of within-person temporal dynamics and the prediction of subsequent GEs.

Compared to prior studies that examined single-point associations or broad daily averages [14,32], this study’s high-resolution, time-aligned modeling [33] offers a more granular view of within-day glucose dynamics. Unlike studies that focused solely on dietary records or controlled feeding conditions [31,34], this analysis highlights hunger as a proximal, low-burden behavioral marker for anticipating glycemic instability in everyday settings.

Theoretically, this study advances a paradigm that conceptualizes metabolic regulation as a dynamic, bidirectional process in which subjective behavioral states may act as proximal indicators of behaviors (eg, eating) that contribute to downstream metabolic changes [20]. This perspective moves beyond traditional models that treat glucose control as an automatic or isolated process, emphasizing instead the reciprocal interplay between behavior and physiology. This conceptualization aligns with dual-process models of appetite regulation, which highlight the integration of homeostatic and hedonic signals in guiding eating behavior and metabolic outcomes [35,36].

### Limitations

Several limitations in this study should be noted. Hunger was assessed using a single binary item, which may not fully capture the complexity of appetite or eating drive. Furthermore, hunger was analyzed as an isolated behavioral signal, although it is likely influenced by unmeasured contextual factors such as stress, emotional state, and the surrounding food environment. Individual differences in interoceptive awareness and self-report accuracy could also influence results. The sample consisted primarily of women and may not generalize to other populations. Given that some sex-related differences in glucose levels were observed, future studies with more balanced sex representation are needed to clarify whether sex meaningfully moderates these associations. Importantly, food intake was not measured, limiting our ability to directly examine the relationship between reported hunger and subsequent eating behavior, especially when eating can occur without the presence of hunger [37]. Therefore, the observed association between hunger and subsequent GEs is likely mediated by unmeasured eating behavior rather than reflecting a direct physiological effect of hunger itself. Additionally, repeated EMA prompts may have induced reactivity, potentially influencing participants’ awareness of hunger and their self-reports. Finally, the models did not include an explicit residual correlation structure for repeated measures over time. As a result, temporal autocorrelation may not have been fully accounted for. Despite these limitations, this study offers novel insight into the dynamic interplay between behavioral states and glucose regulation in daily life and underscores the importance of considering subjective experience in metabolic research. Future studies should consider multidimensional hunger assessments, incorporate dietary assessment, and include more diverse samples to improve generalizability, and explore how such momentary signals can be operationalized to guide real-time interventions that support metabolic health across diverse populations.

### Conclusions

In conclusion, this study highlights hunger as a dynamic and actionable behavioral signal that bridges subjective experience and physiological response. By integrating real-time hunger assessments with CGM data, these findings provide a foundation for enhancing personalized strategies to reduce metabolic risk in overweight populations.

## Supplementary material

10.2196/85897 Checklist 1 STROBE checklist.

## Abbreviations

CGM: continuous glucose monitoring
EMA: ecological momentary assessment
GE: glucose excursion
JITAI: just-in-time adaptive interventions

## References

[1] Kashiwagi K, Inaishi J, Kinoshita S (2023). Assessment of glycemic variability and lifestyle behaviors in healthy nondiabetic individuals according to the categories of body mass index. PLoS One.

[2] Salkind SJ, Huizenga R, Fonda SJ, Walker MS, Vigersky RA (2014). Glycemic variability in nondiabetic morbidly obese persons: results of an observational study and review of the literature. J Diabetes Sci Technol.

[3] Ye L, Gu W, Chen Y (2020). The impact of shift work on glycemic characteristics assessed by CGM and its association with metabolic indices in non-diabetic subjects. Acta Diabetol.

[4] Fysekidis M, Cosson E, Banu I, Duteil R, Cyrille C, Valensi P (2014). Increased glycemic variability and decrease of the postprandial glucose contribution to HbA1c in obese subjects across the glycemic continuum from normal glycemia to first time diagnosed diabetes. Metabolism.

[5] Monnier L, Colette C, Owens DR (2008). Glycemic variability: the third component of the dysglycemia in diabetes. Is it important? How to measure it?. J Diabetes Sci Technol.

[6] Monnier L, Mas E, Ginet C (2006). Activation of oxidative stress by acute glucose fluctuations compared with sustained chronic hyperglycemia in patients with type 2 diabetes. JAMA.

[7] Polonsky WH, Henry RR (2016). Poor medication adherence in type 2 diabetes: recognizing the scope of the problem and its key contributors. Patient Prefer Adherence.

[8] Wangnoo SK, Sethi B, Sahay RK, John M, Ghosal S, Sharma SK (2014). Treat-to-target trials in diabetes. Indian J Endocrinol Metab.

[9] Kim YI, Choi Y, Park J (2023). The role of continuous glucose monitoring in physical activity and nutrition management: perspectives on present and possible uses. Phys Act Nutr.

[10] Lemmens SG, Martens EA, Kester AD, Westerterp-Plantenga MS (2011). Changes in gut hormone and glucose concentrations in relation to hunger and fullness. Am J Clin Nutr.

[11] Patti AM, Giglio RV, Ciaccio M (2025). New frontiers in nutritional and therapeutic interventions for obesity phenotypes. Medicina (Kaunas).

[12] de Wit M, van Raalte DH, van den Berg K (2023). Glucose variability and mood in people with type 1 diabetes using ecological momentary assessment. J Psychosom Res.

[13] Shiffman S, Stone AA, Hufford MR (2008). Ecological momentary assessment. Annu Rev Clin Psychol.

[14] Naguib MN, Hegedus E, Raymond JK (2022). Continuous glucose monitoring in adolescents with obesity: monitoring of glucose profiles, glycemic excursions, and adherence to time restricted eating programs. Front Endocrinol (Lausanne).

[15] Hegedus E, Salvy SJ, Wee CP (2021). Use of continuous glucose monitoring in obesity research: a scoping review. Obes Res Clin Pract.

[16] Guerra S, Sparacino G, Facchinetti A, Schiavon M, Man CD, Cobelli C (2011). A dynamic risk measure from continuous glucose monitoring data. Diabetes Technol Ther.

[17] Tronieri JS, Wadden TA, Walsh O (2020). Effects of liraglutide on appetite, food preoccupation, and food liking: results of a randomized controlled trial. Int J Obes (Lond).

[18] Pannicke B, Blechert J, Reichenberger J, Kaiser T (2022). Clustering individuals’ temporal patterns of affective states, hunger, and food craving by latent class vector-autoregression. Int J Behav Nutr Phys Act.

[19] Ehrmann D, Schmitt AJ, Rubertus P, Kulzer B, Hermanns N (2020). 783-P: Can mood and energy levels be predicted by preceding glucose values? Combining ecological momentary assessment (EMA) and continuous glucose monitoring (CGM). Diabetes.

[20] Rethorst CD, Githinji P, Seguin-Fowler RA, MacMillan Uribe AL, Szeszulski J, Liao Y (2023). Real-time assessment of the bidirectional relationship between affective states and glucose: protocol for a 14-day observational study. JMIR Res Protoc.

[21] (2000). Obesity: preventing and managing the global epidemic. Report of a WHO consultation. World Health Organ Tech Rep Ser.

[22] Vigers T, Chan CL, Snell-Bergeon J (2019). cgmanalysis: an R package for descriptive analysis of continuous glucose monitor data. PLoS One.

[23] Barhak J Visualization and pre-processing of intensive care unit data using Python data science tools. https://www.modsimworld.org/papers/2018/MODSIM_2018_Barhak.pdf.

[24] VanderPlas J (2016). Python Data Science Handbook: Essential Tools for Working with Data.

[25] Harris CR, Millman KJ, van der Walt SJ (2020). Array programming with NumPy. Nature.

[26] Hunter JD (2007). Matplotlib: a 2D graphics environment. Comput Sci Eng.

[27] Waskom ML (2021). seaborn: statistical data visualization. J Open Source Softw.

[28] Blundell JE, Finlayson G, Gibbons C, Caudwell P, Hopkins M (2015). The biology of appetite control: do resting metabolic rate and fat-free mass drive energy intake?. Physiol Behav.

[29] Kim J, Lam W, Wang Q (2019). In a free-living setting, obesity is associated with greater food intake in response to a similar premeal glucose nadir. J Clin Endocrinol Metab.

[30] Matabuena M, Pazos-Couselo M, Alonso-Sampedro M, Fernández-Merino C, González-Quintela A, Gude F (2023). Reproducibility of continuous glucose monitoring results under real-life conditions in an adult population: a functional data analysis. Sci Rep.

[31] Blaak EE, Antoine JM, Benton D (2012). Impact of postprandial glycaemia on health and prevention of disease. Obes Rev.

[32] Jarvis PR, Cardin JL, Nisevich-Bede PM, McCarter JP (2023). Continuous glucose monitoring in a healthy population: understanding the post-prandial glycemic response in individuals without diabetes mellitus. Metabolism.

[33] Matabuena M, Sartini J, Gude F (2024). Multilevel functional data analysis modeling of human glucose response to meal intake. arXiv.

[34] Hall H, Perelman D, Breschi A (2018). Glucotypes reveal new patterns of glucose dysregulation. PLoS Biol.

[35] Lowe MR, Butryn ML (2007). Hedonic hunger: a new dimension of appetite?. Physiol Behav.

[36] Berthoud HR (2011). Metabolic and hedonic drives in the neural control of appetite: who is the boss?. Curr Opin Neurobiol.

[37] Schembre SM, Liao Y, Huh J, Keller S (2020). Using pre-prandial blood glucose to assess eating in the absence of hunger in free-living individuals. Eat Behav.

